# Association of fat mass profile with natriuretic peptide receptor alpha in subcutaneous adipose tissue of medication-free healthy men: A cross-sectional study

**DOI:** 10.12688/f1000research.14198.2

**Published:** 2018-07-18

**Authors:** Petros C. Dinas, Eleni Nintou, Dimitra Psychou, Marnie Granzotto, Marco Rossato, Roberto Vettor, Athanasios Z. Jamurtas, Yiannis Koutedakis, George S. Metsios, Andreas D. Flouris

**Affiliations:** 1 FAME Laboratory, Department of Exercise Science, University of Thessaly, Trikala, Greece; 2 Institute of Sport, Faculty of Education Health and Wellbeing, University of Wolverhampton, Walsall, UK; 3 School of Physical Education and Exercise Science, University of Thessaly, Trikala, Greece; 4 Department of Medicine – DIMED, Internal Medicine 3, University of Padova, Padova , Italy

**Keywords:** NPRA, atrial natriuretic peptide, lipolysis, BMI, obesity

## Abstract

**Background: **Atrial natriuretic peptide increases lipolysis in human adipocytes by binding to natriuretic peptide receptor-A (NPRA). The aim of the current study was to examine the associations of NPRA mRNA of subcutaneous adipose tissue with fat mass, fat-free mass, body mass index (BMI) and arterial blood pressure in medication-free healthy men.

**Method: **Thirty-two volunteers [age (years): 36.06±7.36, BMI: 27.60±4.63 (kg/m ^2^)] underwent assessments of body height/weight, % fat mass, fat-free mass (kg), blood pressure, and a subcutaneous adipose tissue biopsy via a surgical technique.

**Results:** We found that NPRA mRNA was negatively associated with % fat mass (r=-0.40, R ^2^=0.16, p=0.03) and BMI (r=-0.45, R ^2^=0.20, p=0.01). Cohen’s *f ^2^* effect size analyses showed a small effect size between NPRA mRNA and BMI ( *f ^2^*=0.25). One-way analysis of variance with Bonferroni post-hoc tests showed a tendency for mean differences of NPRA mRNA across BMI categories (p=0.06). This was confirmed by Cohen’s *d* effect size analyses revealing a large effect size of NPRA mRNA between obese individuals (BMI≥30 kg/m ^2^) and either normal weight (BMI=19-25 kg/m ^2^; *d*=0.94) or overweight (BMI=25-30 kg/m ^2^; *d*=1.12) individuals.

**Conclusions:** NPRA mRNA is negatively associated with % fat mass and BMI in medication-free healthy men, suggesting a possible role of NPRA in the control of fat mass accumulation.

## Introduction

Atrial natriuretic peptide (ANP) lowers arterial pressure to maintain fluid volume homeostasis, thus protecting against renal and cardiac pathogenesis ^1^. ANP also increases lipolysis in human adipocytes ^2^ by binding to natriuretic peptide receptor-A (NPRA) ^3^. NPRA is less expressed in subcutaneous adipose tissue (SAT) in obese individuals and type 2 diabetes patients than in normal glucose tolerant individuals ^4^. Also, NPRA signalling in skeletal muscle may enhance long-term insulin sensitivity ^5^. Collectively, NPRA may potentially treat obesity-related disorders while ANP may play a role in the therapeutic mechanisms of beta-adrenoceptor antagonists in the mitigation of heart dysfunction and utilization of lipid mobilization ^6^. However, the role of ANP in lipolysis has been primarily investigated mainly *in vitro* models ^7– 9^, in human blood cells from individuals under medication treatment ^10^, and in animal models ^9^. To our knowledge, no such information is currently available in relation to the role of its receptor (NPRA) on the adipocytes of healthy individuals. Therefore, the aim of the current study was to examine the associations of NPRA mRNA of SAT with fat mass, fat-free mass (FFM), body mass index (BMI) and arterial blood pressure (BP) in medication-free healthy men.

## Methods

The study was approved by the Ethics Committee of the University of Thessaly (protocol no. 698/2013). The inclusion criteria were: healthy adult men, non-smokers, no chronic disease and/or being under medication treatment. The participants were recruited by advertisements in a local newspaper in Trikala, Thessaly, Greece and the data collection started in July 2013 and ended in June 2014. Written consent was obtained from the 32 healthy men recruited [age (years): 36.06±7.36, BMI: 27.60±4.63 (kg/m ^2^)].

To avoid misleading results, the participants refrained from exercise, alcohol, and passive smoking 72h prior the measurements, while they followed an overnight fast before they visit the physiology laboratory in the School of Exercise Science between 07:00–09:00 am. PCD and DP performed the following measurements: body height using a Seca 220 (Hamburg, Germany) stadiometer, body weight using a precision scale (KERN & Sohn GmbH, Version 5.3, Germany) and blood pressure (BP) using a Standard Aneroid sphygmomanometer (Medisave, UK) according to standard guidelines ^11^. Briefly, participants were instructed to empty their bladders and sit for five minutes in a relaxed back rest position without talking. BP readings were taken twice, each two minutes apart, while the mean of the two BP readings was considered as the final BP values. Fat mass percentage (%FM) and FFM were measured via bioelectrical impedance using a body composition monitor (Fresenius Medical Care AG & Co. KGaA D-61346 Bad Hamburg, Germany).

Following the aforementioned measurements, the participants underwent a SAT biopsy in the physiology laboratory by a trained physician, as previously described ^12^. Briefly, the site of the incision was disinfected and a 10 ml of 2% xylocaine (no adrenaline) was injected for local anaesthesia. An incision of 2–2.5 cm was made 3–5 cm to the left of the navel. Nearly 500 mg of subcutaneous adipose tissue was captured and removed. The NPRA mRNA analysis of SAT samples is described elsewhere ^13^. Briefly, total RNA was extracted using RNeasy Lipid Tissue mini kit (QIAGEN). First-strand cDNAs were synthesized from equal amounts of total RNA using random primers and M-MLV reverse transcriptase (Promega). Quantitative real-time polymerase chain reaction was performed using Sybr Green fluorophore. 18S rRNA gene was used as a reference for normalization.

Following previous methodology, we removed two mean values (i.e. outliers) of NPRA mRNA that were at a distance of more than two standard deviations from the mean of the distribution ^14, 15^. Also, there were three missing values in the NPRA mRNA analysis of SAT samples due to failure to extract RNA from adipose tissue. Eventually, 27 NPRA mRNA values were included in the statistical analysis using SPSS (version 24; SPSS Inc., Chicago, IL, USA). Normal distribution was determined using Shapiro-Wilk test, whereas Pearson’s correlation coefficient, linear regression, and Cohen’s *f ^2^* effect size (R ^2^/1-R ^2^) ^16^ were used to detect associations between NPRA mRNA, %FM, FFM (kg), BMI, and BP. We also used one-way analysis of variance (ANOVA) with Bonferroni post-hoc tests, and Cohen’s *d* effect size analyses to explore the mean differences of NPRA mRNA across different BMI categories [normal weight <25 kg/m ^2^ (n=9); overweight 25–30 kg/m ^2^ (n=9); obese >30 kg/m ^2^ (n=9)]. The level of significance was set at p≤0.05.

## Results

**Figure 1.**
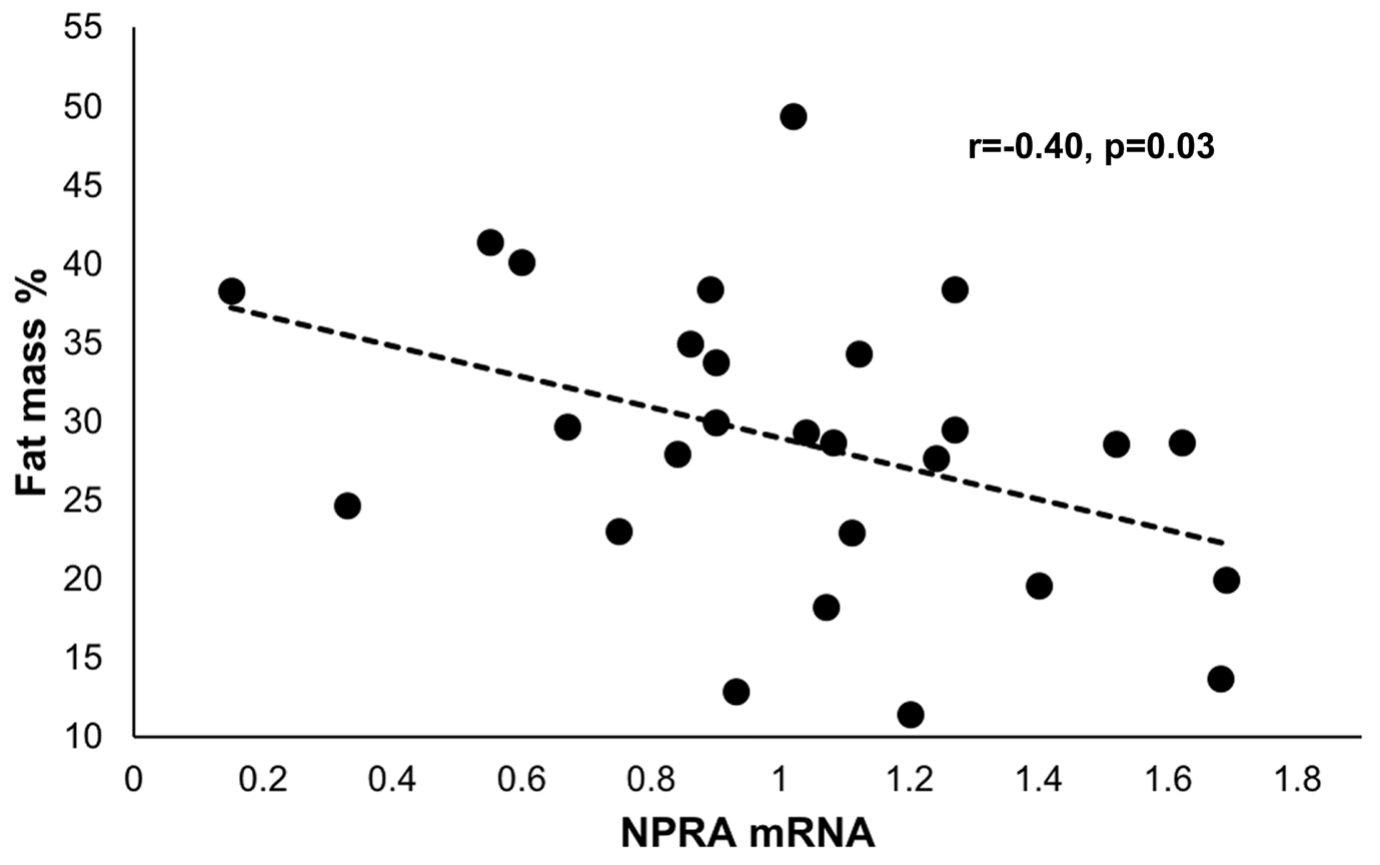
Association of NPRA mRNA with fat mass %.

**Figure 2.**
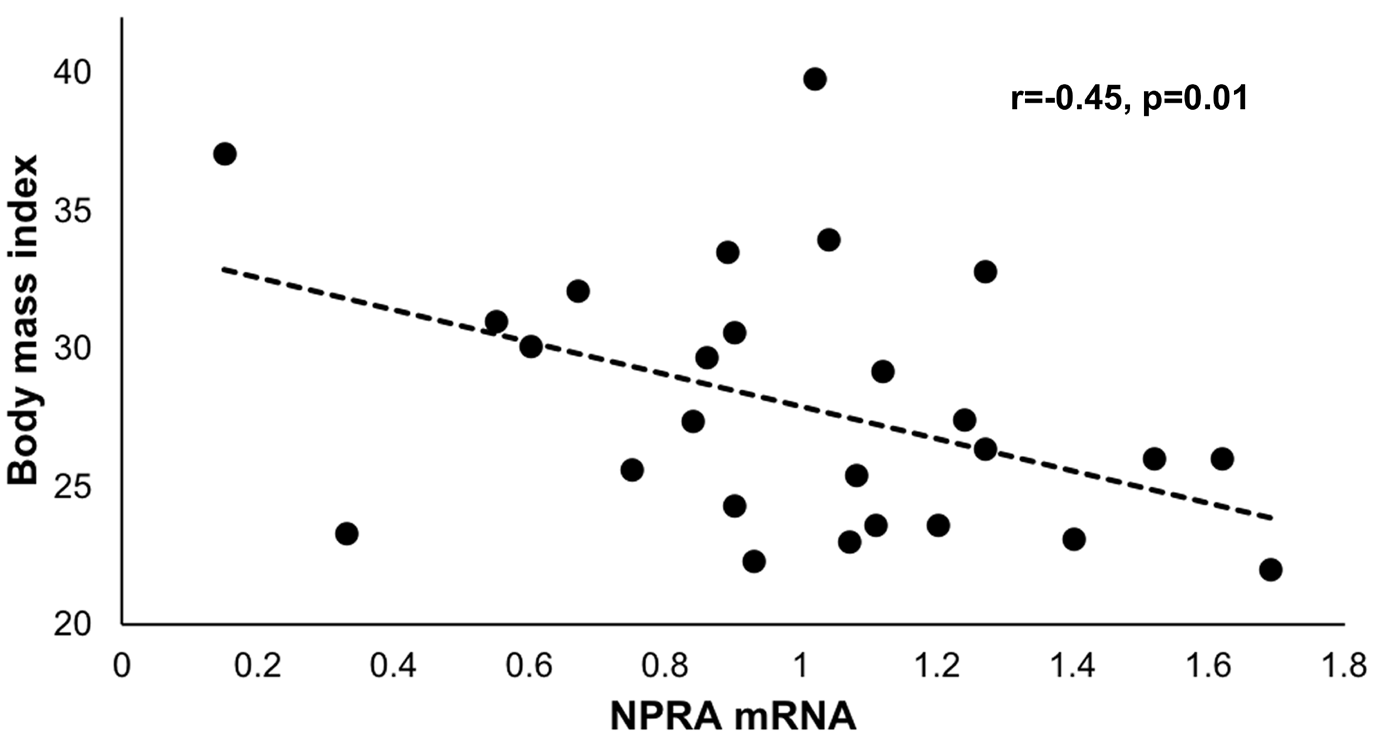
Association of NPRA mRNA with body mass index.

The participants’ characteristics are provided in Table 1. The NPRA mRNA was negatively correlated with %FM (r=-0.40, p=0.03) ( Figure 1) and BMI (r=-0.45, p=0.01) ( Figure 2). No associations were found between NPRA mRNA and FFM, systolic or diastolic BP (p>0.05).

**Table 1.** Characteristics of the participants.

Age (years) (n=32)	36.06±7.36
BMI (kg/m ^2^) (n=32)	27.60±4.62
Fat mass (%) (n=32)	28.32±8.87
Fat free mass (kg) (n=32)	52.90±5.02
Systolic blood pressure (mmHg) (n=32)	124.28±10.09
Diastolic blood pressure (mmHg) (n=32)	84.28±6.91

BMI: Body mass index

Linear regression analyses confirmed the associations between NPRA mRNA and %FM (R ^2^=0.16, p=0.03) as well as BMI (R ^2^=0.20, p=0.01). Cohen’s *f ^2^* effect size analyses showed a small effect size between NPRA mRNA and BMI ( *f ^2^*=0.25), however, no effect size was detected between NPRA mRNA and %FM ( *f ^2^*<0.20). ANOVA demonstrated a strong tendency for mean differences in NPRA mRNA across BMI categories (p=0.06). This was confirmed by Cohen’s *d* effect size analyses in NPRA mRNA, revealing large effect sizes between obese individuals (BMI≥30 kg/m ^2^) and either normal weight (BMI <25 kg/m ^2^; *d*=0.94) or overweight (BMI=25–30 kg/m ^2^; *d*=1.12) individuals.

Subcutaneous adipose tissue NPRA mRNA of medication-free healthy men BMI=Body mass index; SBP=Systolic blood pressure; DBP=Diastolic blood pressure; FFM=Fat-free mass; NPRA= Natriuretic peptide receptor-A. BMI categories= 1. <25 kg/m ^2^, 2. 25–30 kg/m ^2^, 3. >30 kg/m ^2^. Click here for additional data file. Copyright: © 2018 Dinas PC et al. 2018 Data associated with the article are available under the terms of the Creative Commons Zero "No rights reserved" data waiver (CC0 1.0 Public domain dedication).

## Discussion and conclusions

We have shown that the NPRA mRNA is negatively associated with %FM and BMI in medication-free healthy men and that it is expressed less in obese compared to lean individuals. Previous evidence showed that NPRA mRNA is expressed less in normal glucose tolerant individuals than in type 2 diabetes patients ^4^, while it is positively associated with insulin sensitivity ^4^. Given that insulin sensitivity is negatively associated with excessive FM in humans ^17, 18^ the inverse association of NPRA mRNA with %FM and BMI observed in the current study suggests a possible role of NPRA in lowering FM in humans. Indeed, it has been established that natriuretic peptides by binding to NPRA, increase cyclic guanosine monophosphate – a well-known intracellular second messenger – which phosphorylates protein kinase G leading to activation of hormone sensitive lipase ^19, 20^. This process mediates triglyceride degradation (i.e. lipolysis), which subsequently increases fatty acid availability ^19^. This lipolytic effect of NPRA however, appears to be supressed by NPRC action in obese individuals and clearance of NPR3 gene – coding for NPRC – in animals showed to restore NPRA action ^9^, which should be considered in future studies. Furthermore, findings in mice showed that the lack of NPRA gene may increase FM ^9^. Finally, NPRA signalling as part of ANP/NPRA axis may induce a browning of white adipocytes, indicating increased energy expenditure and thus, a potential to mitigate obesity ^21^.

The current study may be affected by methodological limitations such as the lack of insulin sensitivity measurements and *a priori* power calculation to determine the sample size. However, a post-measurements power calculation was conducted using an online software (DSS Research) to test statistical power. This revealed 89% of statistical power for the 27 available samples, based on the NPRA mRNA value (1.02±0.38) we detected in our study and expected NPRA mRNA value (0.81±0.08) from a previous similar study that examined NPRA in SAT in humans ^4^. Another limitation was the lack of triglyceride, cholesterol and ANP plasma levels, to determine whether there is a link with NPRA mRNA, given the association of the aforementioned factors with lipolysis ^2, 19^. Previous evidence also suggests that large adipocytes express higher NPRA mRNA than small adipocytes, indicating enhanced ANP-stimulated lipolysis in large adipocytes ^22^. However, we did not determine the size of the examined adipocytes and its possible association with NPRA mRNA. Furthermore, brain natriuretic peptide (BNP) may alter expression of NPRA to release free fatty acids from adipose tissue, while obesity is inversely associated with circulating BNP, a situation known as “natriuretic handicap” ^23, 24^. Circulating BNP was not measured in our study to examine whether there is an association with NPRA mRNA. Also, ANP may inhibit the secretion of adipokines and cytokines from adipose tissue and, thus, may attenuate chronic inflammation and insulin resistance ^25, 26^, a mechanism that was not investigated in our study. Given the action of ANP/BNP on lipolytic hormones, it would have been interesting to determine hormone sensitive lipase in the current study ^2, 6^. Finally, it is important to note that the current results are limited to the Greek population and male participants, therefore, they should be treated with caution when applied to other ethnicities and females.

In conclusion, NPRA mRNA is negatively associated with %FM and BMI in medication-free healthy men, suggesting a possible role of ANP/NPRA axis in the control of FM accumulation. To date, the investigation of NPRA has mainly focused either on circulating and muscle NPRA ^27– 29^ or on medication-dependent NPRA measurements ^4, 10^. Our study indicates that NPRA may also play role in FM profile of healthy individuals, which should be further explored in a cause-and-effect research setting.

## Data availability

The data referenced by this article are under copyright with the following copyright statement: Copyright: © 2018 Dinas PC et al.

Data associated with the article are available under the terms of the Creative Commons Zero "No rights reserved" data waiver (CC0 1.0 Public domain dedication). 

Dataset 1: Subcutaneous adipose tissue NPRA mRNA of medication-free healthy men 10.5256/f1000research.14198.d197694 ^30^

BMI=Body mass index; SBP=Systolic blood pressure; DBP=Diastolic blood pressure; FFM=Fat-free mass; NPRA= Natriuretic peptide receptor-A.

BMI categories= 1. <25 kg/m ^2^, 2. 25–30 kg/m ^2^, 3. >30 kg/m ^2^.
