# Supplementary material for: Association of fat mass profile with natriuretic peptide receptor alpha in subcutaneous adipose tissue of medication-free healthy men: A cross-sectional study
Source: F1000Res. 2018 Jul 18;7:327. Originally published 2018 Mar 15. [Version 2] doi: 10.12688/f1000research.14198.2 (PMC6053697; doi:10.12688/f1000research.14198.2)
Supplement: Peer review history

# Peer review: Referee response for version 2

**DOI:** 10.5256/f1000research.17059.r36193

**Published:** 2018-07-24

**Authors:** Kailash N. Pandey

**Affiliations:** 1 Department of Physiology, School of Medicine, Tulane University Health Sciences Center, New Orleans, LA, USA

The manuscript has been revised according to the previous comments and there is no further concerns with current manuscript.

I have read this submission. I believe that I have an appropriate level of expertise to confirm that it is of an acceptable scientific standard.

# Peer review: Referee response for version 2

**DOI:** 10.5256/f1000research.17059.r36192

**Published:** 2018-07-19

**Authors:** Marcelo Roberto Choi

**Affiliations:** 1 School of Pharmacy and Biochemistry, University of Buenos Aires, Buenos Aires, Argentina

The manuscript has been revised according to the reviewers' comments. I have no further concerns.

I have read this submission. I believe that I have an appropriate level of expertise to confirm that it is of an acceptable scientific standard.

# Peer review: Referee response for version 1

**DOI:** 10.5256/f1000research.15445.r34721

**Published:** 2018-06-25

**Authors:** Kailash N. Pandey

**Affiliations:** 1 Department of Physiology, School of Medicine, Tulane University Health Sciences Center, New Orleans, LA, USA

The current manuscript by P.C. Dinas et al., describes the association of fat mass profile with the mRNA levels of natriuretic peptide receptor-A (NPRA) in the subcutaneous adipose tissues (SAT) of medication-free healthy individuals. The major goal of the present study was to determine the association of NPRA mRNA in SAT with fat mass (FM), fat-free mass (FFM), body mass index (BMI), and arterial blood pressure in 32 volunteers with average age of 36 years. The subcutaneous adipose tissues biopsy was done by surgical procedures. The authors report that NPRA mRNA was negatively associated with % FM as well as BMI. Based on the present findings, the authors suggest that NPRA signaling mechanism might play a critical role in the control of fat mass accumulation. The results of the current study are interesting and critical in understanding the versatility of the NPRA signaling mechanisms in the health and disease; however, there are some concerns, which need to be considered to improve the contents of this manuscript.

1. The present study establishes a link between NPRA mRNA, % FM, and BMI. The results indicate that atrial and brain natriuretic peptides (ANP, BNP) and their cognate receptor NPRA might play a vital role in lipolysis. Thus, it could have been a logical extension to determine the hormone-sensitive lipases. Although, ANP and BNP have been shown to act as the lipolytic hormones and also to affect energy use and metabolism in adipocytes, thus their actions seem to be associated with hormonal stimulation of lipases (Sengenes et al., 2003 ^1^; Lafontan et al., 2008 ^2^). It would be helpful that these issues and limitation might be discussed in the manuscript. 
2. The ethnicity of the Greek subjects under the study should be indicated and if there is any limitation that could be mentioned. The study is based on the small number of subjects and the limitations might also be indicated.
3. The cGMP- dependent protein kinase 1 (CGK1) phosphorylates perilipin-1, a hormonal-regulated lipase that initiates lipolysis (Sengenes et al., 2003; Lafontan et al., 2005 ^3^). Nonetheless, it has been suggested that the potent lipolytic function of ANP and BNP seems to be restricted to primates (Sengenes et al., 2002 ^4^; Engeli et al., 2012 ^5^). Intriguing was the finding that a lower concentrations of ANP and BNP seem to be associated with obesity, insulin resistance, and metabolic consequences, thereby, ANP-BNP/NPRA/cGMP axis might regulate fat oxidation to prevent obesity and glucose intolerance (Wang et al., 2004 ^6^; Wang et al., 2007 ^7^; Mitsuishi et al., 2008 ^8^; Miyashita et al., 2009 ^9^). These paradigms and issues of the ANP/NPRA/cGMP signaling could be discussed.   
4. Interestingly, ANP-BNP/NPRA signaling axis has been shown to induce a browning of white adipocytes in humans, which seems to be physiologically significant (Enerback, 2010 ^10^; Collins et al., 2014 ^11^; Liu et al., 2018 ^12^). A brief discussion on this aspect of ANP/NPRA signaling mechanisms might be helpful.
5. The previous findings have suggested that NPRA acts as a determinant of insulin sensitivity; however, the upregulation of natriuretic peptide receptor-C (NPRC) decreases the glucose tolerance in obase subjects, which seems to repress ANP-BNP/NPRA signaling axis and thereby the lipolytic effects of ANP was completely rescued in *Npr3* (coding for NPRC) gene-knockout mice (Bordicchia et al., 2012 ^13^). The significance of these previous findings and the relationship to the current work could be discussed in the current manuscript.  
6. The conclusions of the present study are based on the findings in male gender. The limitations on the inclusion of female gender should be mentioned.

 

 I have read and reviewed the manuscript and believe that I have the expertise to evaluate and state that the current work is of an acceptable scientific quality.  Indeed, there are some concerns in the manuscript, which are summarized above.

I have read this submission. I believe that I have an appropriate level of expertise to confirm that it is of an acceptable scientific standard.

## References

[1] SengenesCBouloumieAHaunerHBerlanMBusseRLafontanMGalitzkyJ: Involvement of a cGMP-dependent pathway in the natriuretic peptide-mediated hormone-sensitive lipase phosphorylation in human adipocytes. *J Biol Chem*.2003;278(49) : 10.1074/jbc.M303713200 48617-26 10.1074/jbc.M303713200 12970365

[2] LafontanMMoroCBerlanMCrampesFSengenesCGalitzkyJ: Control of lipolysis by natriuretic peptides and cyclic GMP. *Trends Endocrinol Metab*.19(4) : 10.1016/j.tem.2007.11.006 130-7 10.1016/j.tem.2007.11.006 18337116

[3] LafontanMMoroCSengenesCGalitzkyJCrampesFBerlanM: An unsuspected metabolic role for atrial natriuretic peptides: the control of lipolysis, lipid mobilization, and systemic nonesterified fatty acids levels in humans. *Arterioscler Thromb Vasc Biol*.2005;25(10) : 10.1161/01.ATV.0000183728.14712.d8 2032-42 10.1161/01.ATV.0000183728.14712.d8 16123323

[4] SengenèsCZakaroff-GirardAMoulinABerlanMBouloumiéALafontanMGalitzkyJ: Natriuretic peptide-dependent lipolysis in fat cells is a primate specificity. *Am J Physiol Regul Integr Comp Physiol*.2002;283(1) : 10.1152/ajpregu.00453.2001 R257-65 10.1152/ajpregu.00453.2001 12069952

[5] EngeliSBirkenfeldALBadinPMBourlierVLoucheKViguerieNThalamasCMontastierELarrouyDHarantIde GlisezinskiILieskeSReinkeJBeckmannBLanginDJordanJMoroC: Natriuretic peptides enhance the oxidative capacity of human skeletal muscle. *J Clin Invest*.2012;122(12) : 10.1172/JCI64526 4675-9 10.1172/JCI64526 23114600PMC3533552

[6] WangTJLarsonMGLevyDBenjaminEJLeipEPWilsonPWVasanRS: Impact of obesity on plasma natriuretic peptide levels. *Circulation*.2004;109(5) : 10.1161/01.CIR.0000112582.16683.EA 594-600 10.1161/01.CIR.0000112582.16683.EA 14769680

[7] WangTJLarsonMGKeyesMJLevyDBenjaminEJVasanRS: Association of plasma natriuretic peptide levels with metabolic risk factors in ambulatory individuals. *Circulation*.2007;115(11) : 10.1161/CIRCULATIONAHA.106.655142 1345-53 10.1161/CIRCULATIONAHA.106.655142 17339551

[8] MitsuishiMMiyashitaKItohH: cGMP rescues mitochondrial dysfunction induced by glucose and insulin in myocytes. *Biochem Biophys Res Commun*.2008;367(4) : 10.1016/j.bbrc.2008.01.017 840-5 10.1016/j.bbrc.2008.01.017 18194668

[9] MiyashitaKItohHTsujimotoHTamuraNFukunagaYSoneMYamaharaKTauraDInuzukaMSonoyamaTNakaoK: Natriuretic peptides/cGMP/cGMP-dependent protein kinase cascades promote muscle mitochondrial biogenesis and prevent obesity. *Diabetes*.2009;58(12) : 10.2337/db09-0393 2880-92 10.2337/db09-0393 19690065PMC2780866

[10] EnerbäckS: Human brown adipose tissue. *Cell Metab*.2010;11(4) : 10.1016/j.cmet.2010.03.008 248-52 10.1016/j.cmet.2010.03.008 20374955

[11] CollinsSSarzaniRBordicchiaM: Coordinate control of adipose 'browning' and energy expenditure by β-adrenergic and natriuretic peptide signalling. *Int J Obes Suppl*.2014;4(Suppl 1) : 10.1038/ijosup.2014.6 S17-20 10.1038/ijosup.2014.6 27152160PMC4850585

[12] LiuDCeddiaRPCollinsS: Cardiac natriuretic peptides promote adipose 'browning' through mTOR complex-1. *Mol Metab*.2018;9: 10.1016/j.molmet.2017.12.017 192-198 10.1016/j.molmet.2017.12.017 29396369PMC5870104

[13] BordicchiaMLiuDAmriEZAilhaudGDessì-FulgheriPZhangCTakahashiNSarzaniRCollinsS: Cardiac natriuretic peptides act via p38 MAPK to induce the brown fat thermogenic program in mouse and human adipocytes. *J Clin Invest*.2012;122(3) : 10.1172/JCI59701 1022-36 10.1172/JCI59701 22307324PMC3287224

# Response

**Published:** 2018-07-14

**Authors:** Petros Dinas

**Affiliations:** FAME Laboratory, University of Thessaly, Greece

We thank you very much for your comments. Please see below our responses to your comments:

Comment 1: Thank you for this suggestion. Indeed, this would have been an interesting analysis to perform. Unfortunately, there are no samples available at this stage to perform the suggested work. Therefore, we have included in the limitations (Page 6, paragraph 2) the lack of hormone sensitive lipase measurements.

Comment 2:  We have added this information in the limitations (Page 6, paragraph 2).

Comment 3:  The issue regarding the obesity prevention via ANP/BNP axis has been added in the Discussion section (Page 6, paragraph 1).  Insulin sensitivity measurements in the current study has also been indicated as a limitation. (Page 6, paragraph 2).

Comment 4:  We have added this information at the end of the first paragraph of the Discussion section (Page 6, paragraph 1).

Comment 5:  We have included a brief discussion on these topics in the first paragraph of the Discussion section (Page 6, paragraph 1).

Comment 6: We have added this information in the limitations (Page 6, paragraph 2).

# Peer review: Referee response for version 1

**DOI:** 10.5256/f1000research.15445.r33152

**Published:** 2018-04-24

**Authors:** Marcelo Roberto Choi

**Affiliations:** 1 School of Pharmacy and Biochemistry, University of Buenos Aires, Buenos Aires, Argentina

The present work by Dinas et al. is a descriptive study that investigated the association of NPRA mRNA of subcutaneous adipose tissue (SAT) with fat mass, fat-free mass, body mass index (BMI) and arterial blood pressure in thirty-two medication-free healthy volunteers. The authors observed a negative association between NPRA mRNA with %fat mass and BMI. Based on these results the authors suggest a possible role of ANP-NPRA axis in the control of FM accumulation. Although the topic is of interest, some issues need to be addressed in order to improve the quality of the manuscript.

1. Given that NPRA plays a role in lipolysis, it would have been interesting if the authors could determine the levels of triglycerides and cholesterol as well as ANP plasma levels in order to verify a possible correlation between these parameters and the percentage of fat and/or NPRA mRNA levels in SAT. This issue should be considered in the discussion section and mentioned as a limitation of this study.
2. Please provide more details about blood pressure registers (for example: number of measures, guideline used to define hypertension, brand of the device used).
3. Yu et al. ^1^ suggested that adipocyte size is an important determinant of ANP-stimulated lipolysis. In this way, expressed higher mRNA levels of receptor (NPR)-A and hormone sensitive lipase as well as more NPR-A on the membrane than small It would have been interesting if the authors had determined the NPRA mRNA according to the size of the adipocyte. This issue should be considered in the discussion section and mentioned as a limitation of this study. (see also Laurencikiene et al. ^2^)
4. It has been reported that obesity is associated with low circulating levels of BNP and recent evidences suggest an altered expression of BNP receptors-both the signaling receptors (NPR)-A and the clearance NPR-C receptor-in Pivovarova et al. ^3^; Gentili et al. ^4^). Moreover, increased tissue secretion of adipokines and cytokines has been implicated in the chronic low-grade state associated with obesity (Schindler et al. ^5^; Moro et al. ^6^). This issue should be considered in the discussion section and mentioned as a limitation of this study.

I have read this submission. I believe that I have an appropriate level of expertise to confirm that it is of an acceptable scientific standard, however I have significant reservations, as outlined above.

## References

[1] YuJYuHCKimKAKwonKBParkJWKimSZKimSHParkBH: Differences in the amount of lipolysis induced by atrial natriuretic peptide in small and large adipocytes. *J Pept Sci*.2008;14(8) : 10.1002/psc.1035 972-7 10.1002/psc.1035 18383440

[2] LaurencikieneJSkurkTKulytéAHedénPAströmGSjölinERydénMHaunerHArnerP: Regulation of lipolysis in small and large fat cells of the same subject. *J Clin Endocrinol Metab*.2011;96(12) : 10.1210/jc.2011-1702 E2045-9 10.1210/jc.2011-1702 21994963

[3] PivovarovaOGögebakanÖKlötingNSparwasserAWeickertMOHaddadINikiforovaVJBergmannAKruseMSeltmannACBlüherMPfeifferAFRudovichN: Insulin up-regulates natriuretic peptide clearance receptor expression in the subcutaneous fat depot in obese subjects: a missing link between CVD risk and obesity?. *J Clin Endocrinol Metab*.2012;97(5) : 10.1210/jc.2011-2839 E731-9 10.1210/jc.2011-2839 22419733

[4] GentiliAFrangioneMRAlbiniEVaccaCRicciMADe VuonoSBoniMRondelliFRotelliLLupattelliGOrabonaC: Modulation of natriuretic peptide receptors in human adipose tissue: molecular mechanisms behind the "natriuretic handicap" in morbidly obese patients. *Transl Res*.186: 10.1016/j.trsl.2017.06.001 52-61 10.1016/j.trsl.2017.06.001 28651075

[5] SchindlerTHCardenasJPriorJOFactaADKreisslMCZhangXLSayreJDahlbomMLicinioJSchelbertHR: Relationship between increasing body weight, insulin resistance, inflammation, adipocytokine leptin, and coronary circulatory function. *J Am Coll Cardiol*.2006;47(6) : 10.1016/j.jacc.2005.10.062 1188-95 10.1016/j.jacc.2005.10.062 16545651

[6] MoroCKlimcakovaELolmèdeKBerlanMLafontanMStichVBouloumiéAGalitzkyJArnerPLanginD: Atrial natriuretic peptide inhibits the production of adipokines and cytokines linked to inflammation and insulin resistance in human subcutaneous adipose tissue. *Diabetologia*.2007;50(5) : 10.1007/s00125-007-0614-3 1038-47 10.1007/s00125-007-0614-3 17318625

# Response

**Published:** 2018-07-14

**Authors:** Petros Dinas

**Affiliations:** FAME Laboratory, University of Thessaly, Greece

We thank you very much for your comments. Please see below our responses to your comments:

Comment 1:  Thank you for this suggestion. Indeed, this would have been a very interesting analysis to perform. Unfortunately, there are no samples available at this stage to perform the suggested work. Therefore, we have included this information in the limitations of the Discussion section (Page 6, paragraph 2).

Comment 2:  We have added further details about blood pressure registers and we provide a relevant reference (24) in methods section (Page 4, paragraph 1).

Comment 3:  As suggested, we have included this information in the limitations of the Discussion section and we provide the relevant reference (25). (Page 6, paragraph 2).

Comment 4:  As suggested, we have added this information in the limitations along with the relevant references (26,27, 28,29). (Page 6, paragraph 2).
